# Center of Mass Estimation for Impaired Gait Assessment Using Inertial Measurement Units

**DOI:** 10.1109/TNSRE.2023.3341436

**Published:** 2024-01-12

**Authors:** Gabrielle C. Labrozzi, Holly Warner, Nathaniel S. Makowski, Musa L. Audu, Ronald J. Triolo

**Affiliations:** Department of Biomedical Engineering, Case Western Reserve University, Cleveland, OH 44106 USA; Motion Study Laboratory, Veterans Affairs Medical Center, Louis Stokes Cleveland Department, Cleveland, OH 44106 USA; Department of Biomedical Engineering, Case Western Reserve University, Cleveland, OH 44106 USA; Motion Study Laboratory, Veterans Affairs Medical Center, Louis Stokes Cleveland Department, Cleveland, OH 44106 USA; Lakeland Community College, Kirtland, OH 44094 USA; MetroHealth Medical Center, Department of Physical Medicine and Rehabilitation, Cleveland, OH 44109 USA; Motion Study Laboratory, Veterans Affairs Medical Center, Louis Stokes Cleveland Department, Cleveland, OH 44106 USA; Department of Biomedical Engineering, Case Western Reserve University, Cleveland, OH 44106 USA; Motion Study Laboratory, Veterans Affairs Medical Center, Louis Stokes Cleveland Department, Cleveland, OH 44106 USA; Department of Biomedical Engineering, Case Western Reserve University, Cleveland, OH 44106 USA; Motion Study Laboratory, Veterans Affairs Medical Center, Louis Stokes Cleveland Department, Cleveland, OH 44106 USA

**Keywords:** Body-mounted sensors, center of mass, inertial measurement units, pathological gait

## Abstract

Injury or disease often compromise walking dynamics and negatively impact quality of life and independence. Assessing methods to restore or improve pathological gait can be expedited by examining a global parameter that reflects overall musculoskeletal control. Center of mass (CoM) kinematics follow well-defined trajectories during unimpaired gait, and change predictably with various gait pathologies. We propose a method to estimate CoM trajectories from inertial measurement units (IMUs) using a bidirectional Long Short-Term Memory neural network to evaluate rehabilitation interventions and outcomes. Five non-disabled volunteers participated in a single session of various dynamic walking trials with IMUs mounted on various body segments. A neural network trained with data from four of the five volunteers through a leave-one-subject out cross validation estimated the CoM with average root mean square errors (RMSEs) of 1.44cm, 1.15cm, and 0.40cm in the mediolateral (ML), anteroposterior (AP), and inferior/superior (IS) directions respectively. The impact of number and location of IMUs on network prediction accuracy was determined via principal component analysis. Comparing across all configurations, three to five IMUs located on the legs and medial trunk were the most promising reduced sensor sets for achieving CoM estimates suitable for outcome assessment. Lastly, the networks were tested on data from an individual with hemiparesis with the greatest error increase in the ML direction, which could stem from asymmetric gait. These results provide a framework for assessing gait deviations after disease or injury and evaluating rehabilitation interventions intended to normalize gait pathologies.

## I. Introduction

Walking mobility is important for navigating daily life and promoting a healthy body and productive lifestyle [1]. Compared to other movements, the whole-body contributes and impacts the gait cycle, in which the upper extremities maintain postural stability and the lower body assists in energy conservation and propulsion [2]. In non-disabled individuals, walking exhibits a smooth, cyclic behavior that is both efficient and effortless [2]. However, injury or disease can change the gait cycle in varying degrees. For instance, individuals with Parkinson’s Disease experience freezing of gait [3] with reduced gait speed [4]. Hemiparesis or spinal cord injury may increase gait variability [5] and asymmetry [6], [7] and reduce speed. A strong correlation exists between gait impairments and the quality of life [8] and independence [4]. Thus, numerous rehabilitative and assistive interventions and technologies, including exoskeletons [9], functional neuromuscular stimulation [10], and virtual reality [11] are being developed to improve walking after neuro-musculoskeletal disease or injury.

While studies have explored joint kinematics [12], ground reaction forces [13], and spatiotemporal parameters [14] as indicators of walking impairment or rehabilitation progress, center of mass (CoM) kinematics are global parameters that indicate the quality of gait and motor control of walking. The CoM is a point about which the mass of the body is equally distributed in all directions so that when external forces are applied, only linear acceleration occurs [15]. Just as all parts of the body contribute to walking, CoM is a simplification of whole-body movement in space. Thus, CoM has been widely used to describe the body’s movement during the gait cycle [16] where its trajectories follow well-defined sinusoidal patterns in non-disabled individuals [17] and is associated with dynamic stability [16]. When observing pathological gait including individuals with Parkinson’s Disease [18], hemiparesis [19], or spinal cord injuries [20], CoM trajectories deviate significantly from neurotypical patterns.

CoM kinematics have successfully been applied in robot bipedal locomotion control, which motivates the exploration of similar control systems for assistive devices or rehabilitation programs designed to stabilize locomotion in individuals with pathologic gait. However, unlike other signals such as joint angles, CoM kinematics cannot be measured directly in humans and must be estimated computationally from mass parameters of biomechanical models or other potentially more efficient methods. In the field of robotics, CoM position has been derived from joint angles [21], force sensing insoles [22], inverted pendulum models [23], and knowledge of all segment masses and lengths [21], [22], [23]. These estimation methods are accurate, but challenging to translate to human walking. Unlike bipedal robots, human segment lengths and masses are difficult to measure accurately, and external instrumentation like encoders to capture joint trajectories are awkward and inconvenient to don and doff. Furthermore, the inverted pendulum model commonly used in the study of bipedal robot locomotion assumes the length of the pendulum is fixed, which does not translate to human applications as joints flex and extend to reduce energy expenditure. For studies of human locomotion, CoM kinematics have been estimated using motion capture data [24] or ground reactions from force plate data via numerical integration [25], which are restricted to laboratory settings and unsuitable for clinical applications.

Inertial measurement units (IMUs) offer a promising alternative to estimating CoM kinematics due to their small size, affordability [26], reliability [27] and wireless communication capabilities. A previous study conducted by Cardarelli et al. [28] proposed a single IMU estimation approach by removing gravitational acceleration and integrating using a fourth-order Runge-Kutta procedure. The approach accurately estimated CoM trajectories in the mediolateral (ML), anteroposterior (AP) and inferior/superior (IS) directions but has been validated only in non-disabled treadmill walking. Even though a treadmill provides a constant walking speed for repeatability, the inconsistent walking speeds present in pathological gait [29] may give rise to estimation errors. Other studies have used IMU signals to train neural networks and estimate CoM. Betker et al. [30] proposed a feedforward backpropagation ANN utilizing two accelerometers with one located on the trunk and the other on the shank. The network accurately estimated CoM in the AP and IS directions but projected higher errors in the ML direction. Furthermore, estimates did not capture oscillating changes that may occur in the AP direction and focused on the single support phase of gait rather than the continuous trajectory of the CoM throughout the entire gait cycle. Chebel and Tunc [31] proposed a multilayer feedforward backpropagation neural network using signals from five IMUs to predict CoM movements. Even though accurate predictions were attained from non-disabled volunteers, the estimator required inclusion of joint angle measurements derived from sensors mounted precisely on the shoulder, hips, and trunk, which might prove to be impractical for routine clinical application. Furthermore, without information from the lower extremities such a limited set of sensors may be subject to errors resulting from increased variability and abnormal movements that occur during pathologic gait.

This study proposes a neural network that estimates CoM trajectories from a set of wearable IMUs during the full gait cycle. Unlike other approaches, which focus on estimating CoM with non-disabled gait data, our approach is intended to function accurately both during neurotypical dynamics and simulated pathological conditions. We further characterize how the number and location of selected IMUs influence estimation accuracy, thereby advancing definition of an optimal network with a minimal sensor set for a simplified setup and application to individuals with movement disorders.

## II. Methods

### A. Experimental Procedure

Five healthy volunteers (3 men and 2 women; age: 27.4±5.5yrs.; height: 178.2±9.4cm; weight: 66.9±7.9kg; Table I), labelled AB01, AB02, etc. to AB05, participated in a single session of walking experiments to generate data for training the neural networks. Each volunteer was screened to ensure they had no known medical or acute orthopedic problems; and they all signed a written consent form approved by the Institutional Review Board of the Louis Stokes Cleveland Veterans Affairs Medical Center (number 1591730, approved 3/11/2021).

**Fig. 1.**
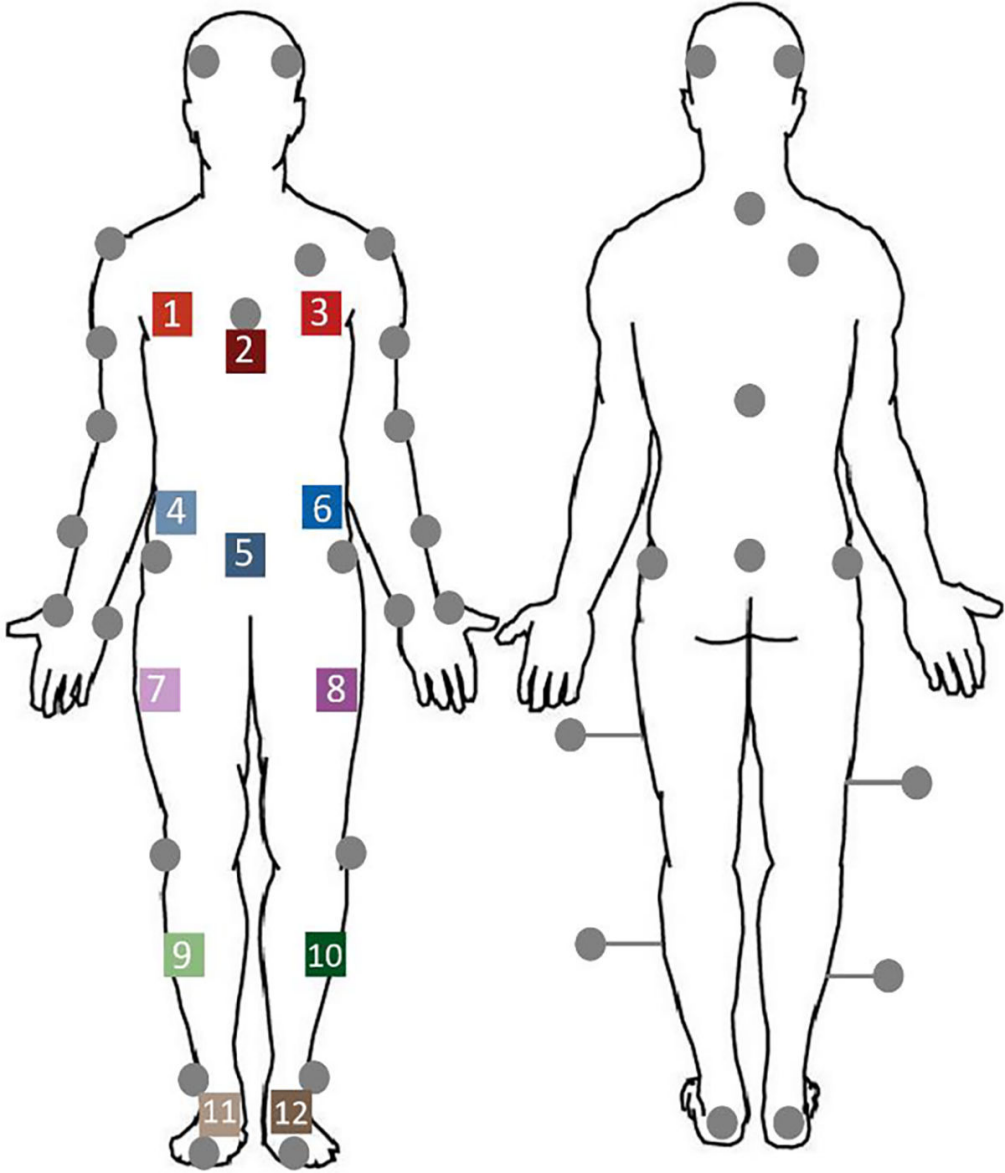
Position of the body-mounted sensors. The gray circles are the reflective markers and the 12 color squares are the Xsens IMUs. The IMUs were positioned on each lower extremity segment and the trunk. While not shown, each IMU had a reflective marker attached above the sensor’s origin point.

Each participant wore a 38-reflective marker set and 12 IMUs with three on the thoracic region, three on the mid abdomen, one positioned anterolaterally on each thigh and shank, and one per foot (Fig. 1). The location of the IMUs ensured that CoM estimates included contributions from both the torso and lower body [2]. The sensors were placed in arbitrary locations and in random orientations to generalize the donning process. Specific locations and orientations on the limb were not prescribed to obviate the need to maintain a precise placement methodology. IMUs were securely taped to the skin to limit excessive movement. The reflective markers generated positional data through a 16-camera motion capture system (VICON, Oxford Metrics, Oxford, UK) at 100Hz. The IMUs (Xsens MVN; Xsens Technologies B.V, Netherlands) captured triaxial linear acceleration, angular velocity, and magnetic field strength data at 60Hz. Although not shown in Fig. 1, additional reflective markers were attached to each IMU [32] to capture their mounting locations. For some trials, a walker was employed to mimic the assistive device used by individuals with movement disorders. The walker’s height was adjusted based on each participant’s height and preference.

**Fig. 2.**
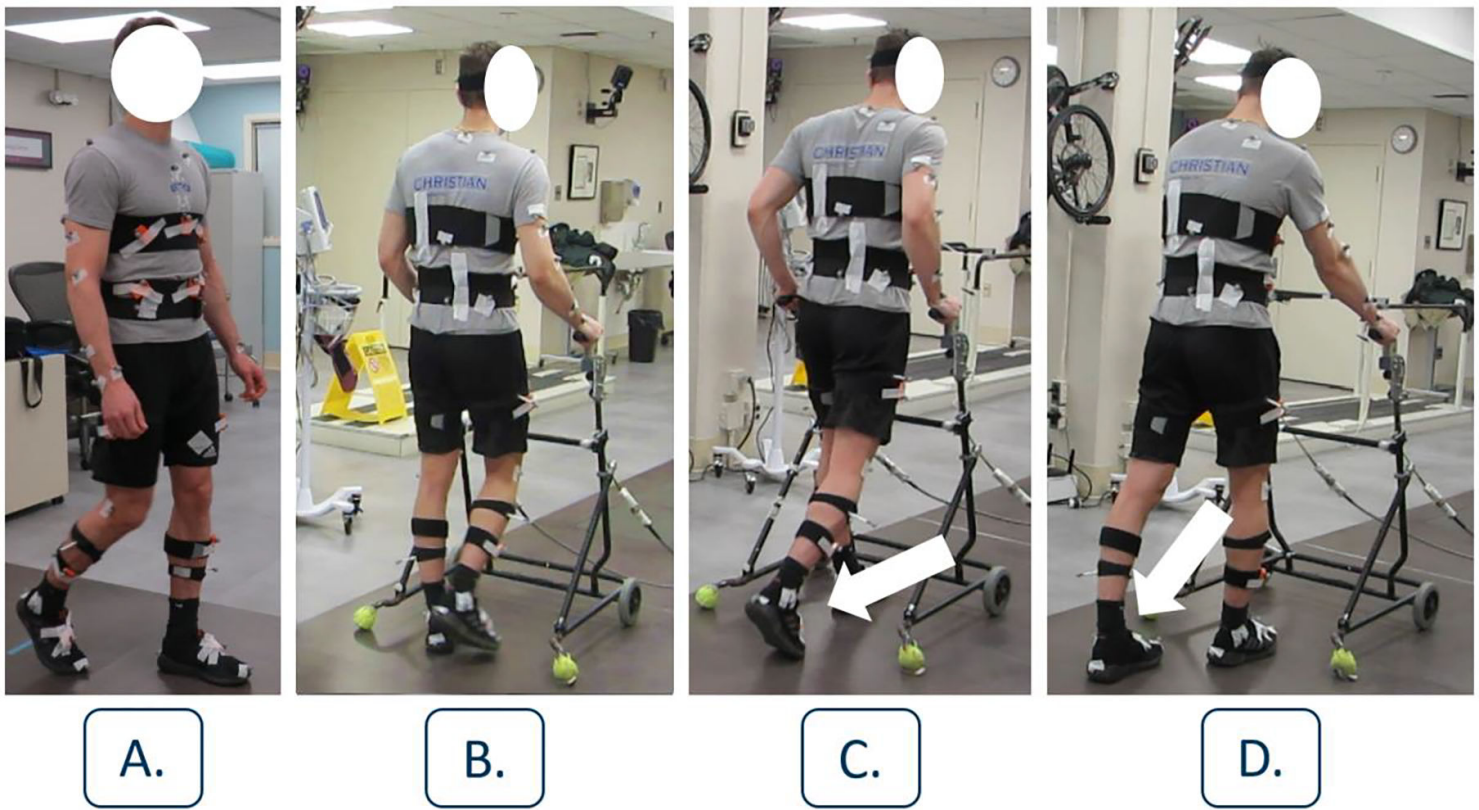
Each participant completed walking trials under various gait dynamics. Each condition had subjectively normal and slow pace trials. A) Neurotypical gait dynamics. B) Continuous pattern with walker. C, D) Discontinuous pattern with a pause during the DSP to push the walker forward. Step initiation with plantarflexion (C) and without (D).

Each volunteer completed dynamic and static trials. For eight static trials, each participant held various poses: standing, lying supine, and on each side for sensor calibration, discussed in Methods Section, II-B. The participants also completed a single T-trial with the arms outstretched and legs together for subject-specific model generation by the VICON software. Gait speed [15], stability [33], and variability [34] influence the CoM kinematics, which are impacted in pathologic gait. Therefore, in each walking trial volunteers walked across a 10m walkway at subjectively normal and slow paces under four conditions (Fig. 2): (A) walking with neurotypical gait without an assistive device (15 trials); (B) walking continuously with a walker as if pushing a shopping cart (20 trials); and (C, D) walking using a walker with a discontinuous stepping pattern (11 trials each). Conditions C and D involved pausing during the double support phase (DSP) to push the walker forward prior to step initiation, a strategy typically adopted by individuals having either minimal plantarflexion ability or using ankle foot orthoses (AFOs). Thus, in Condition C the participants initiated steps with full-range plantarflexion, whereas in Condition D they used minimal or no plantarflexion. The number of dynamic trials varied by condition to ensure at least 100 steady steps were captured. Supplemental Videos show the gait dynamics presented here.

### B. Post-Processing

**Fig. 3.**
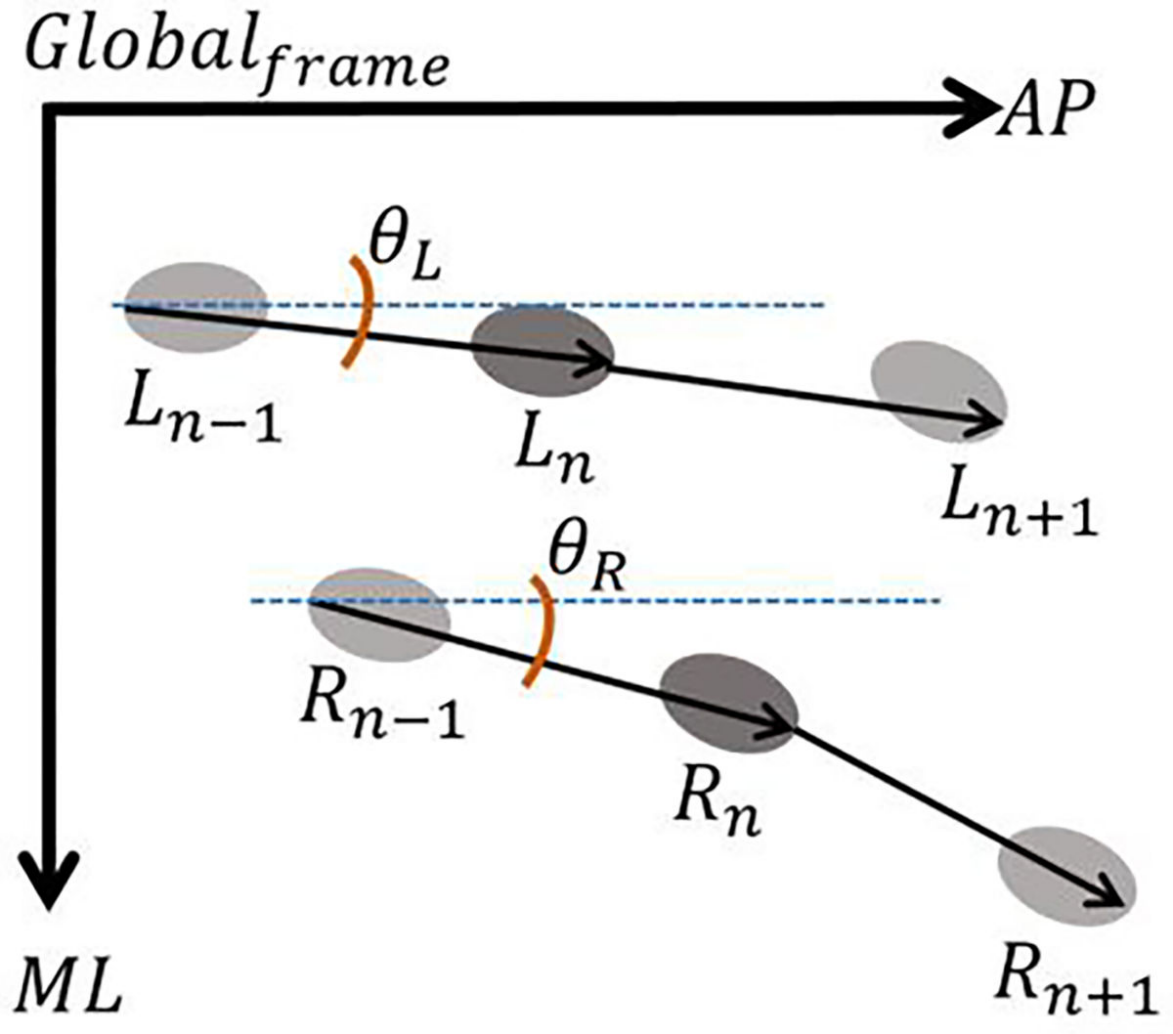
Footstep Position Example. A vector between the stance foot and the preceding one was generated to account for heading and drift from a straight line progression. The position of the right (R) and left (L) foot are with respect to the global frame of the lab. is the angle between the stance foot and the global AP direction.

Sensor data were pre-processed by filtering with a second order lowpass Butterworth filter with a 15Hz cutoff, and then down-sampling the marker data to 60Hz to match the IMU data capture frequency. VICON marker positions and anthropometric tables [35] were used to analytically compute the global, whole-body CoM in each direction. For ease of analysis, CoM position data were referred to a reference frame with the origin at the middle of the support foot during the stance phase of each step [36]. To mitigate CoM errors due to variability in heading and drift from straight line progression, we assumed a flat walking surface and applied a rotation matrix to the CoM signals [37] as shown in equation (1):

(1) $${CoM}_{\text{body}}=\left(\begin{array}{ccc} cos\theta_{R/L} & -sin\theta_{R/L} & 0\\ sin\theta_{R/L} & cos\theta_{R/L} & 0\\ 0 & 0 & 1 \end{array}\right)*\left(\begin{array}{c} {ML}_{\text{global}}\\ {AP}_{\text{global}}\\ {IS}_{\text{global}} \end{array}\right)$$

where $ML,\;AP,\;IS_{\text{global}}$ are the global CoM positions and $\theta_{R/L}$ is the angle between the right or left foot locations in the DSP and the global AP direction (Fig. 3). A vector pointing from the heel to the toe marker for each sequential stance foot was used to compute the angle. We normalized the IS component to the participant’s height, and linearly detrended the AP direction to focus on oscillating changes.

Assuming arbitrary placement of IMUs on each segment, IMU data were transformed from their local reference frames to those of the individual body segments based on VICON data. For this study, we utilized motion capture to register the sensors to the underlying anatomy to eliminate potential sources of error with our proposed method. First, the reference orientation of each body segment was determined via inverse kinematics [38]. Then calibration parameters were computed to transform the sensors’ signals to the body segments’ coordinate frames via the least-squares estimate proposed by Umeyama [39], where the relationship between the acceleration expressed in the segment and sensor frames is defined as:

(2) $$a_{\text{seg}}=c^{*}R^{*}a_{acc}+t$$

where $a_{seg}$ is the acceleration measurement expressed in the segment frame and $a_{acc}$ is the measurement expressed in the sensor frame. For a given accelerometer, c is the scale factor, t is the bias, and R is a rotation matrix relating the sensor frame to the segment frame. Angular velocity and magnetic field strength data were transformed by multiplying by the rotation matrix only.

### C. Neural Network for CoM Estimation

The foundation of the neural network estimation algorithm stemmed from the work of Hnat et al. [40] who employed a feed-forward neural network to estimate CoM during bipedal standing perturbations. Upon initial exploration, this neural network structure failed to estimate CoM for the various walking conditions. Due to step dependency and sequential dynamics of the gait cycle, a Long Short-term Memory (LSTM) neural network layer [36] followed by a bidirectional LSTM (biLSTM) provided more accurate results in our preliminary investigations and were selected for further study.

**Fig. 4.**
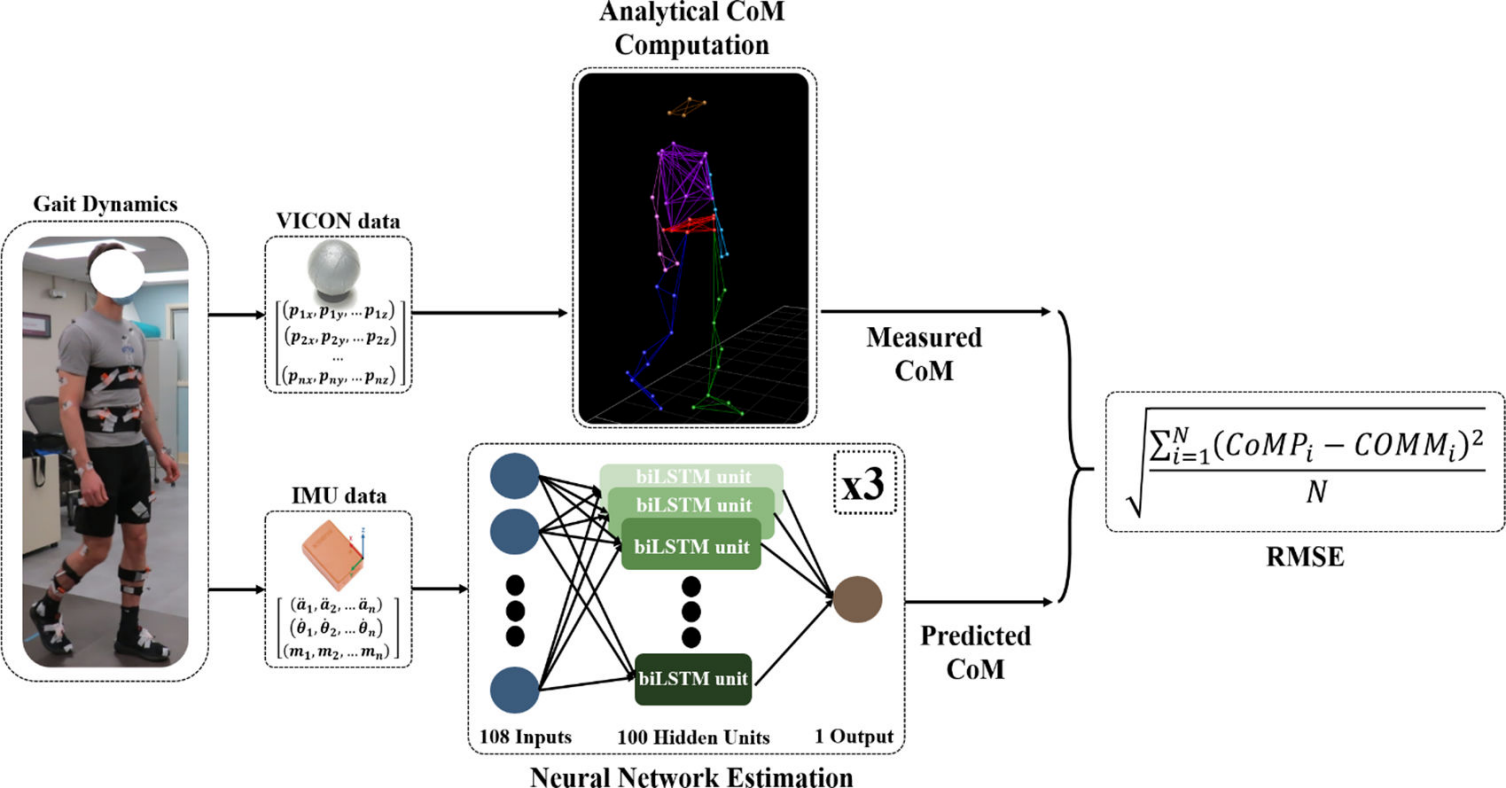
Neural Network Workflow. The VICON data from the gait condition trials is used in conjunction with the anthropometric tables to compute the measured CoM. Each of the three neural networks take the IMU data as the inputs and produces the estimated CoM for one direction, ML, AP, and IS. The estimates are compared to the measured CoM by computing the RMSE where CoMP is the predicted CoM and CoMM is the measured.

Fig. 4 outlines the workflow for the network estimations. Linear acceleration, angular velocity, and magnetic field strength data in all three directions from the IMUs served as inputs into the neural network, making for a total of 108 inputs. We explored using either a single network to output all three CoM components or an independent neural network for each component. The latter generated more accurate estimates; thus we had three networks estimating either the ML, AP or IS CoM position. We compared network estimates with the motion capture CoM which served as the gold standard [40].

Each biLSTM neural network consisted of 5 hidden layers: 1) Sequence Input Layer, 2) Batch Normalization Layer, 3) biLSTM Layer, 4) Fully Connected Layer, 5) Regression Layer. To reduce network initialization sensitivity and ease training optimization, we included a batch normalization layer [41]. For the biLSTM layer, we started training the network with 10 hidden units and continued training in increments of 10 until the validation data’s root mean square error (RMSE) stopped improving. This resulted in a final value of 100 hidden neurons. The regression layer used a half-mean-squared error objective function to minimize error between the measured (CoMM) and predicted (CoMP) CoM signals. An L2 regularization term was added to the cost function, E(θ), to minimize the complexity and reduce overfitting. The L2 regularization term was also adjusted to determine the optimal value for each network. The ML network used an L2 regularization of 0.1 and the AP/IS networks used 0.01. The final cost function was defined as

(3) $$\text{E}(\theta )=\frac{1}{2\;\text{S}}\sum_{\text{i}=1}^{\text{S}}\sum_{\text{j}=1}^{\text{N}}{\left({\text{CoMM}}_{\text{ij}}-{\text{CoMP}}_{\text{ij}}\right)}^{2}+\lambda *\left(0.5{\text{w}}^{\text{T}}*\text{w}\right)$$

where N is total number of responses, S is the sequence length, λ is the L2 regularization parameter, and w is the weight vector [42], [43]. The network was built and trained using the Deep Learning Toolbox of MATLAB 2021a.

A Leave-One-Subject out cross validation (LOSOCV) was used for training and testing the neural networks. During each cross validation session, a random selection of trials from 4 participants (74% of the data) were utilized for internal network training and validation, where it was further separated into 70% for training and 30% for validation. A trial was defined as a collection of steady-state (continuous constant velocity) steps within one pass across the 10m walkway. In addition to Conditions B-D (emulating pathologic gait) we included unimpaired walking (Condition A) in this process since an aim of rehabilitation is to address gait impairments until they better resemble typical and more efficient locomotor patterns. The remainder of trials (from the 4 participants set and all trials from the participant left out) were reserved for testing (26% of all data collected) after correcting for any observed offsets. This process was repeated 5 times with the average RMSE calculated from all LOSOCV comparisons. In the subsequent discussion, testing trials similar to the training data will be referred to as “Group 1”, and the left-out participant’s data set as the “test volunteer” and/or “Group 2”.

Within each training/validation data set, a conventional k-fold cross validation test with a k of 5 was performed [44]. These internal validation tests followed the same procedure set by Hnat et al. [40] in which the trials for each k-fold were shuffled. Each cross-validation’s RMSE was calculated, and the median RMSE network was saved to account for the cost and benefit of each k-fold.

The steps outlined above were repeated with networks retrained and tested with only Condition A trials (unimpaired gait) and then for Conditions B-D (emulated pathologic gait) to determine the ability of such a network to generalize to atypical gait patterns without explicit inclusion in the training dataset.

### D. PCA to Determine Optimal Configuration

Previous studies trained and tested networks on all configurations to determine optimal sensor sets for estimation [45]. However, with 12 sensors this is impractical due to computational time required (4,096 possible configurations). We used Principal Component Analysis (PCA) to efficiently identify independent factors with redundant information and optimally select sensors [46]. First, data from each sensor were fused together with an indirect Kalman filter via the *ahrsfilter* system object in MATLAB to yield individual orientations for all trials and participants. The data from each trial and participant were concatenated and had zero mean for every sensor [47].

The ‘percent variance explained’ method was selected when determining the number of principal components for analysis and was computed with IBM’s SPSS Statistical Software (Version 28). The percent variance-explained method illustrates the number of principal components that explain a cumulative variance of 60–70 percent [48]. The maximum number of iterations was set high at 250 to ensure convergence would be reached; convergence occurred within 10 iterations each time. Assuming the data were at least partially correlated, an oblique rotation, Direct Oblimin, was performed on the eigenvectors to help interpret the PCA factors by creating a simple structure [49], [50], [51]. From the pattern matrix, the number of sensors included in the analysis was optimized to ensure the cross-loading ratio for each sensor did not exceed 75% across the components and the loadings were at least 0.4 to ensure a stable solution and simple structure [52], [53].

The PCA indicated which sensors contained redundant information based on its projection onto the principal components. From the PCA and loading information, sensor configurations were selected for retraining/testing the developed neural networks. The explanations for the configurations are detailed further in the Discussion Section, IV-C. Using the previously defined network parameters, we repeated the evaluation process and saved the RMSEs for comparison.

### E. Statistical Analysis

To explore the accuracy of the trained neural networks, we calculated RMSE when comparing CoMM and CoMP per trial, and the mean and standard deviation (SD) RMSE per testing group. Each testing group consisted of a collection of walking conditions (A-D). We then completed a one-way ANOVA with a Tukey post-hoc test to complete a multiple comparison test and set the error rate for all pairwise comparisons. For the multiple comparison test, we determined if there was a significant difference between the 16 sensor sets, for 720 comparisons- 120 comparisons per Group/CoM direction: 6*((16*(16–1))/2). We used the sensor sets as our independent variable/factors and an alpha level of 0.05 to identify statistical significance. All RMSEs from the testing trials were analyzed in MATLAB.

## III. Results

### A. Neural Network Estimates

**Fig. 5.**
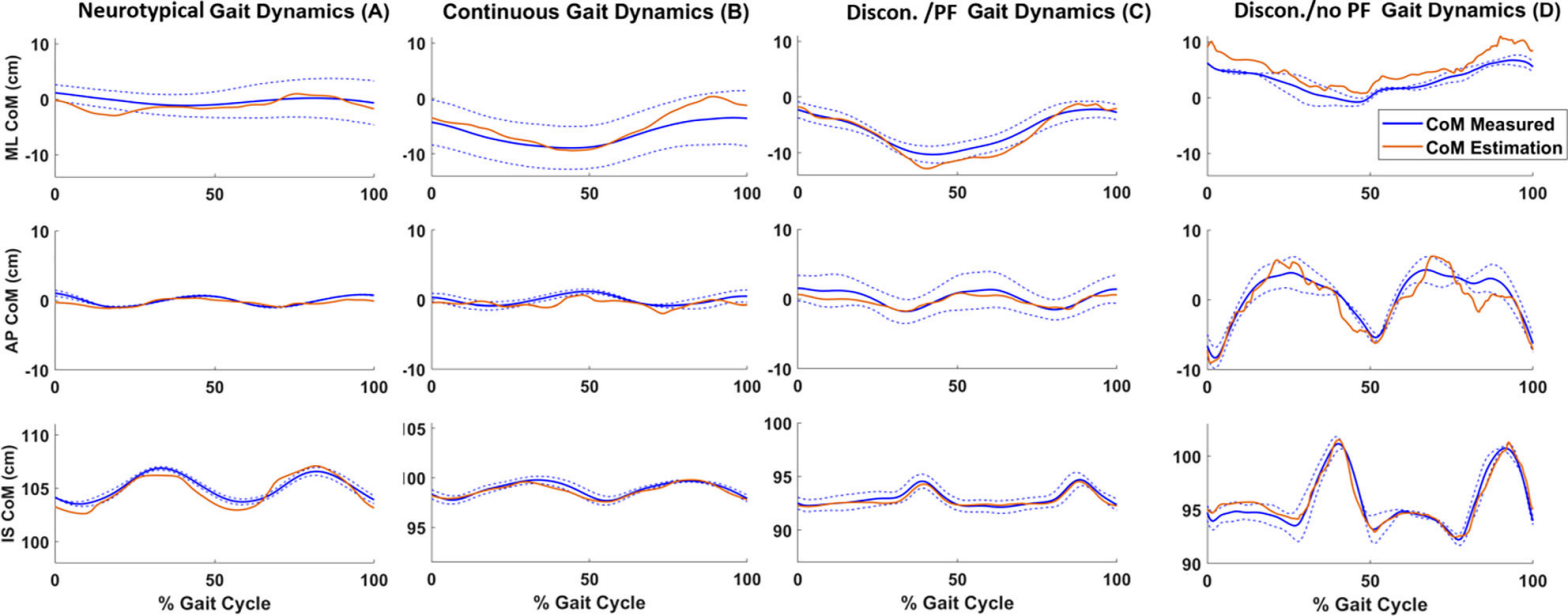
Neural Network estimates. The average analytical CoM trajectory (solid blue), SD (dashed blue), and average network estimates (orange) for the ML, AP, and IS directions respectively. The plots are normalized over a percent gait cycle. The testing trials come from different subjects within testing Group 1. The letters in parentheses correspond to the various conditions outlined in Fig. 2. (Discon. = Discontinuous, PF = plantarflexion).

Fig. 5 depicts the neural network’s CoM estimates for four selected testing trials in the ML (top), AP (middle), and IS (bottom) directions respectively from Group 1. The blue solid and dashed lines are the Mean±SD for the measured CoM trajectories over a stride normalized gait cycle. The orange line is the estimated CoM trajectory from the network. The IS vertical axes display the same range for each condition instead of the same scale since the trials were from different volunteers. The normalized gait cycle initiated at right heel strike. Thus, negative values in the ML direction indicate a trajectory shifting towards the right foot, and positive values represent a shift toward the left foot.

As evident in Fig. 5, the AP and ML profiles for Conditions C and D (paused DSP) partially approached neurotypical patterns with slight non-smooth behavior in the ML direction during DSP. Table II contains the Mean±SD RMSE for the two testing groups for each CoM direction. The network accurately estimated CoM in all 3 directions with the greatest fidelity observed in the IS direction with a RMSE of 0.40 cm and 0.75 cm for Group 1 and 2, respectively. The ML direction resulted in the highest error with a RMSE of 1.44 cm (Group 1) and 2.35 cm (Group 2). When we trained the network with only Condition A trials (neurotypical gait), the predictions achieved a Mean±SD RMSEs of 1.61±0.88 cm and 2.22±0.76 cm for Group 1 and 2 respectively. Then for Conditions B-D, the ML network attained RMSEs of 1.75±0.79 cm and 2.41±0.68 cm for Group 1 and 2.

### B. PCA and Sensor Selection Process

From our PCA, the percent cumulative variance surpassed 70% at the fourth PC; thus, we confined further analysis to a maximum of four PCs. When optimizing the pattern matrix and finding a stable solution, the Left Abdomen sensor was removed from the analysis due to a high cross-loading ratio above the 75% threshold. The Left Foot sensor had a ratio of 75.8% between two PCs. This sensor was kept in the analysis since the ratio was close to the 75% threshold. However, we took this into account when selecting the final candidate configurations.

For the four PCs, the chest sensors were grouped in PC1, shank and feet in PC2, abdominal sensors in PC3 and thighs in PC4. With these observations, we constructed the 15 reduced sensor configurations listed in Table III. Sensor Set #1 included all sensors, and #2 focused on using all sensors from the first 3 PCs, excluding the left abdomen sensor due to its high cross-loading. The feet were removed from Sets #3–16 and medial chest sensor was removed from Sets #3–5, 7, 11–12, and 14–16. For Sensor Set #6, we removed the left shank sensor and #4 explored the impact of focusing on the first three PCs by removing the thigh sensors. We explored lateral contributions to the CoM estimates via Sensor Sets #5, 7, 11, 12, and 14 and medial contributions through sets #8–10 and 13. Assuming perfect left-right symmetry, Sets #9/10 and 11/12 explored estimation accuracies between sensors on the same (right thigh/shank) and opposite legs (left thigh, right shank). Lastly, we explored how accuracy changed when we selected sensors from only two or less PCs with Sets #15 and 16. The neural network was retrained/tested for each sensor set using the previously defined LOSOCV process; the RMSEs are displayed in Table III.

### C. Comparing Estimates for Sensor Configurations

When the networks were tested on Group 1, on average the accuracy of simplified sensor sets did not change compared to Set #1. For the AP CoM, there were no significant differences among the reduced sensor sets. In the ML direction, Sensor Set #16 had a significantly higher error compared to Sets #1, 8, and 10. The average IS RMSE for Sensor Set #16 was significantly higher compared to all Sets, and Sets #5 and 7 significantly improved compared to Set #15.

On average the CoM estimates did not significantly change (2.35 (ML), 1.81 (AP), 0.76 (IS) cm) when comparing the reduced sensor sets to a complete set for the test volunteer. Sensor Set #5, which used six sensors (left chest, right abdomen, right/left thigh and shank) had a significantly higher ML error (2.58 cm) compared to Sensor Sets #3–4, 6–14, and 16. Similarly, all Sensor Sets except #5 had significantly lower ML errors compared to Set #15. Depending on the sensors selected, some reduced sets exhibited significant advantages when estimating ML CoM over others. For instance, Set #6 showed significant improvements compared to Sets #1, 2, 5, 11, 12, and 15 (2.04 cm), and Set #8 compared to Sets #1, 2, 5, and 15 (2.08 cm). In the AP direction, Set #15 had significantly higher errors compared to Set #3 (RMSE 2.14 cm). For the IS estimates, all Sets except for #2 had significantly lower errors compared to Set #15 (RMSE 0.93 cm), and Set #2 had significantly higher errors compared to Sets #3–4, 6–8, 10–11, 13–14, and 16 (RMSE 0.87 cm). For the 4 Sensor Sets, in all directions, there were no significant differences when comparing sets with two sensors on the same versus different legs (9/10 and 11/12), and medially versus diagonally positioned trunk sensors (9/12 and 10/11).

## IV. Discussion

### A. CoM for Various Walking Conditions

When comparing CoM outputs for *Conditions A* and *B*, the addition of an assistive device did not considerably change CoM trajectory in any of the three directions. This is expected since the walker only restrained arm movements and stepping dynamics remained unaffected. Also, the arms have a minimal weight contribution to the body’s overall CoM compared to other body segments, thus, limiting their effect on CoM trajectories. However, there is a decrease in the vertical displacement for Condition B (walker at typical speed), which is commonly observed in individuals with Parkinson’s Disease [18] or hemiparesis [19]. For a discontinuous stepping pattern, there were observable changes in CoM trajectories, especially in the AP and IS directions. This includes abnormal and unsmooth oscillations, which are prominent features in pathological gait. However, the patterns remained relatively symmetrical, which would be expected from non-disabled individuals simulating requested symmetric gait abnormalities. When participants used the assistive device, ML CoM displayed a larger deviation towards the right foot compared to the left (Fig. 5B–D). During the experiment, participants described the walker as having a slight rightwards drift which may have caused the rightward shift in CoM.

### B. Neural Network Estimates

The neural network performed well when estimating CoM from all 12 IMUs for Group 1; the largest error occurred in the ML direction with a RMSE of 1.44 cm. CoM estimation accuracies are comparable to other studies, especially in the AP and IS directions [31]. The ML direction had slightly higher estimation errors, but this could stem from the introduction of the simulated abnormal gait conditions, which increased gait variability. The network’s accuracy declined for Group 2, with the largest error also occurring in the ML direction (2.35 cm RMSE). However, the reduced accuracy still compares favorably to other studies in the AP and IS directions [31]. Even though gait patterns between male and female volunteers may significantly differ [54], there were no noticeable differences if a female was left out versus a male when observing the results across the LOSOCV sessions. Thus, further exploration is required to learn more about the generalization of our approach and the effects of other confounding factors.

Previous studies estimated CoM from neurotypical gait only, so we determined the ability of the network to generalize to atypical gait patterns. When we retrained/retested the networks with only Condition A trials, we observed higher RMSEs for Group 1 surprisingly but lower for Group 2 as expected; thus, partially supporting our conclusion that abnormal gait conditions contributed to higher variability and higher errors in network estimates. The results did not significantly change compared to Conditions A-D, confirming that Condition A trials did not impact the overall estimation accuracy. When we checked Condition B-D’s impact on the network estimations, the results did not significantly change the network’s estimation compared to Condition A’s results and when compared to Conditions A-D.

### C. PCA and Sensor Selection Process

Our PCA indicated a stable solution with three or four PCs. This breakdown was expected based on biomechanics of gait [2]. The trunk, represented by the chest sensors, is part of the passenger unit, whereas the lower extremities compose the locomotor unit, which was monitored by the leg sensors. The abdominal sensors, which are located near the pelvis, involve both the passenger and locomotive aspects.

Each sensor configuration was carefully selected based on gait kinematics, plausible observations in pathological gait, and previous studies. For example, Sensor Set #3 focused on possible lateral rotations in the trunk with the removal of the middle chest sensor. The foot sensors were removed since they provided similar information to that of the shanks. We checked the effect of the cross loading for the left shank sensor with Set #6. Exploring combinations for the thigh/shank sensors was important because asymmetry is prevalent in pathological gait due to varying muscle strength. Ideally, we expected to observe no significant differences in the accuracy or a significantly higher accuracy when using sensors on both extremities.

Lastly, Floor-Westerdijk et al. proposed a CoM estimator based on a single IMU located at the pelvis; thus, we explored how our network compared with Sensor Set #16 [55]. Floor-Westerdijk et al. suggested adding another IMU to capture trunk movements, which is especially important in pathological gait where individuals may use their upper extremities to maintain balance. We tested this observation with Set #15, but retained a shank and chest sensor since pathologic gait is subjected to abnormal leg movements.

### D. CoM Estimates for Sensor Configurations

Group 1’s CoM estimates were more accurate overall compared to Group 2 in all directions, which we expected since Group 2 was an introduced test volunteer. In general, there were no distinct increases in RMSEs for each CoM direction and group as the number of sensors decreased to 3, except for Sets #5 (ML, Group 2) and #2 (IS, Group 2). When we reduced to two or fewer sensors, CoM error increased significantly. This supports our PCA analysis that at least 3 sensors from different grouping of sensors are needed to capture the variation in the CoM signal. Across all sensor sets in both groups, the IS direction had the lowest CoM errors. This was expected since the ML and AP CoMs are prone to more variations between subjects than in the IS direction. This could be due to natural drifting of the direction of progression, inconsistent walking speeds, and the acclimation of various gait conditions. Acclimation may help with consistent gait patterns. Therefore, we mitigated this variability by providing time for acclimation prior to recording the trials and transforming CoM to the body’s reference frame instead of a global frame.

Our long-term goal is to develop a network that accurately estimates all CoM trajectories from a minimal sensor set. Comparing Group 1’s results, Set #16 was the only set with significantly worse accuracy in the ML and IS trajectories. Therefore, we removed this set from further consideration. The higher estimation errors for this set also align with Floor-Westerdijk et al. conclusion that additional sensors on the trunk are recommended for higher accuracy [55]. However, our data suggests that at least 3 sensors are required for higher accuracy since there were significant error increases for Set #15. When exploring the generalization of the network, Sets #2, 5, and 15 had significantly higher errors compared to other sets. In all 3 cases, we used the left chest and right shank sensor data. Thus, this sensor combination is potentially losing information from the CoM trajectories that is critical for estimation accuracy. Accordingly, these sets were removed from our network candidates.

### E. Sensor Configuration Candidates

To select an optimal sensor set, we viewed the results for all directions and testing groups. From the sixteen configurations, Sensor Sets #6, 9, and 13 are the strongest candidates for assessing control and rehabilitation methods in the future since they incorporated a minimal number of sensors and had low RMSEs across all directions. When comparing sets #9 and 10, these two sets differed in the sensor positions on the thighs and shanks. Set #10 had the sensors on the same side whereas Set #9 had them on opposite sides. In non-disabled walking, the gait is relatively symmetrical [56], so we would capture approximately the same amount of information from either side. Thus, either Set is a strong candidate since there were no significant differences between RMSEs. However, when considering populations exhibiting asymmetric gait, we suggest Set #9 as a plausible candidate over Set #10 to collect data from both lower extremities. Further examination of these sets is required to conclude how they perform with asymmetric gait. Set #13 had relatively low RMSEs and no significant errors. The difference between Set #13 and Set #9 was the removal of the thigh sensors, thus relying on a single sensor to capture lower extremity data. These results imply refraining from using only three sensors to characterize asymmetrical gait, but further examination is again required. We plan to explore Sets #6, 9, and 13 in a real-time study with both non-disabled volunteers and persons with gait impairments to inform future outcome assessments.

### F. Limitations of Current Study

One limitation could be the relatively young pool of volunteers in this study. Multiple studies have shown gait variability increases with age [57]. This could potentially decrease estimation accuracy when an older volunteer is introduced. Although the relatively low RMSEs obtained suggest this limitation did not affect our results significantly, further exploration is required to generalize the method and ensure the network’s robustness across age. This project used motion capture for sensor calibration to eliminate potential sources of error in prescribing exact anatomical sensor positions and orientation and generalize the process. The calibration required a set of static trials to transform the sensor data to the respective segmental frame, which may be impractical for routine clinical application. Future implementation would benefit from alternatives to motion capture to register sensor location and orientation to the anatomy; these approaches are being explored in our lab [58] and elsewhere, which we plan to apply in future studies. Lastly, although we trained the network with simulated pathologic gait, the conditions did not cover all possible gait impairments that could be displayed by people with movement disorders, including reduced muscle strength and asymmetry. These factors may impact CoM and the selection of an appropriate sensor suite, and they should be incorporated into future evaluations of this approach.

## V. Performance on Pathological Gait

We also tested the trained neural networks on an individual with post-stroke hemiparesis (male: 58yrs.; height: 162.6cm; weight: 113.4kg) exhibiting observable gait pathologies including toe-drop and step length and time asymmetries. Participant had an average symmetry index [59] of 13.2% and 9.8% for step length and time, and this compares to 2.9% and 5.2% from the study’s non-disabled data. The subject signed the consent form previously mentioned and participated in a single session of walking experiments. The same body-mounted sensors were placed at the locations described above to capture whole-body movement. Twenty trials across the 10m walkway at a self-selected speed were conducted without a walker and fifteen with a walker. Six additional static trials were conducted for sensor calibration.

Data were processed following the same methodology outlined in Section II-B and the results for five sensor combinations are shown in Table IV. These sets were based on our findings and the candidates selected in Section IV-E. Across all sensor sets, the largest error increase compared to our results in Table III occurred in the ML direction. This increase potentially stemmed from the observed asymmetrical gait. Furthermore, when observing the network outputs, the network generally undershot the ML peaks. The individual had a wider step width compared to the training population. This could have contributed to the undershooting of the ML estimates and larger errors. The AP and IS estimates were consistent with Group 2’s results, outlined in Table III, except for Set #9. In the IS direction, Set #1 had a similar RMSE to Group 2’s results, but Sets #6, 9, and 13 observed a larger error increase. When observing Set #6 (IS), #9 (AP, IS), and #13 (IS), the errors partially stemmed from overshooting the peaks. The stroke survivor had a smaller step length compared to the non-disabled population in this study. This could have contributed to the overshooting in Set #9 and impacted this set of sensors more than the others in the AP direction. Further exploration is required to determine the reason for the IS overshooting and determine if this occurrence is consistent across more subjects. Overall, the overall performance of the networks on a data set representing known gait pathologies was encouraging, and further exploration with various gait impairments is warranted.

## VI. Conclusion

CoM kinematics can be estimated accurately in three directions during simulated gait pathologies with biLSTM neural network. 12 IMUs distributed around the torso, thighs and shanks resulted in CoM estimates that matched ground truth values derived from motion capture and segmental mass properties, with estimates in the IS direction exhibiting greatest fidelity. This sensor set was robust and performed equally well on data from a test volunteer uninvolved with network training and internal cross validation. For ease of future implementation with implanted or body worn IMUs, a sensor set consisting of as few as three to as many as five devices can yield accurate predictions of CoM kinematics while walking. Five sensors located on the sternum, medially on the abdomen, left chest, right thigh and left shank exhibited the next most accurate predictions than the full sensor suite across all dimensions. The set of three sensors that included IMUs positioned medially on the abdomen, sternum, and a single sensor on the right shank did not significantly increase CoM errors and exhibited the best efficiency in terms of prediction-to-sensor ratio. Sensor configurations that balanced accuracy and convenience contained four sensors positioned medially on the trunk and abdomen and one sensor on the thigh and shank. Because performance was not significantly different between sensor sets, we recommend considering positioning the thigh and shank sensors on opposite limbs (left thigh and right shank); however, further examination is required to determine if this is necessary and sufficient for asymmetric gait. This analysis needs further validation and real-time implementation in both non-disabled volunteers and persons with motor dysfunction resulting in gait abnormalities in larger trials that control for potential effects of age and gender. Also, a calibration process that does not require motion capture needs to be developed for future clinical implementation. We tested the performance of the neural networks with pathological gait with the smallest error increase in the AP direction compared to the non-disabled data. The network tended to overshoot the IS estimates and undershoot ML estimates. While further exploration is required with more subjects, the results are promising for implementation with pathological gait. This report provides an initial foundation for estimating the CoM with a reduced sensor set for use in controlling gait assist devices and evaluating rehabilitation outcomes.

## Supplementary Material

supp2-3341436

supp4-3341436

supp3-3341436

supp1-3341436

## Figures and Tables

**TABLE I:** Participant Information

Subject	Gender	Age (yrs)	Height (cm)	Weight (kg)

ABOI	M	37	187.9	69.8
AB 02	M	26	184.2	76.2
AB 03	F	24	165.1	55.8
AB 04	M	24	181.6	70.3
AB 05	F	26	172.1	62.8

**TABLE II:** Neural Network Estimates

CoM Profile	Group 1 RMSE (cm)	Group 2 RMSE (cm)

ML	1.44 ± 0.65	2.35 ± 0.67
AP	1.15 ± 0.80	1.80 ± 1.33
IS	0.40 ± 0.17	0.75 ± 0.42

**TABLE III:** CoM Estimates for Each Sensor Configuration

	Sensor Set	Sensor												# Sensor	ML RMSE (cm)		AP RMSE (cm)		IS RMSE (cm)	

															Group 1 (*)	Group 2 (*)	Group 1	Group 2 (*)	Group 1 (*)	Group 2 (*)
		
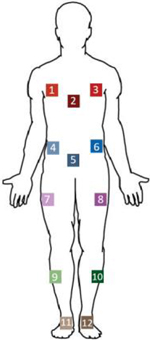	1	1	2	3	4	5	6	7	8	9	10	11	12	12	1.44±0.65	2.35±0.67	1.15±0.80	1.81±1.33	0.40±0.17	0.76±0.42
	2	1	2	3	4	5				9	10	11	12	9	1.62±0.67	2.36±0.72	1.10±0.77	2.03±1.34	0.37±0.15	**0.87±0.45**
	3	1		3	4	5		7	8	9	10			8	1.55±0.69	2.27±0.75	1.08±0.79	1.70±1.26	0.36±0.14	0.65±0.37
	4	1		3	4	5				9	10			6	1.58±0.80	2.11±0.76	1.12±0.80	1.87±1.34	0.39±0.15	0.71±0.40
	5			3	4			7	8	9	10			6	1.54±0.69	**2.58±1.06**	1.11±0.78	1.90±1.17	**0.35±0.15**	0.77±0.41
	6		2	3		5		7			10			5	1.61±0.69	**2.04±0.62**	1.10±0.81	1.91±1.37	0.39±0.15	0.74±0.49
	7			3	4			7		9	10			5	1.54±0.72	2.27±0.59	1.09±0.77	2.08±1.35	**0.35±0.15**	0.70±0.40
	8		2			5				9	10			4	1.51±0.69	**2.08±0.80**	1.13±0.77	1.99±1.30	0.37±0.15	0.69±0.41
	9		2			5			8	9				4	1.55±0.76	2.18±0.73	1.09±0.76	1.91±1.22	0.39±0.17	0.75±0.44
	10		2			5		7		9				4	1.49±0.65	2.21±0.81	1.10±0.86	2.04±1.28	0.37±0.16	0.67±0.39
	11			3	4			7		9				4	1.63±0.75	2.29±0.68	1.15±0.78	2.05±1.33	0.36±0.15	0.73±0.36
	12			3	4				8	9				4	1.61±0.77	2.28±0.70	1.08±0.82	1.98±1.25	0.38±0.18	0.76±0.42
	13		2			5				9				3	1.64±0.73	2.21±0.68	1.14±0.84	1.90±1.27	0.39±0.18	0.69±0.43
	14			3	4					9				3	1.61±0.66	2.29±0.71	1.06±0.75	1.95±1.29	0.39±0.16	0.71±0.33
	15			3						9				2	1.63±0.84	**2.74±1.16**	1.15±0.82	**2.14±1.50**	0.45±0.21	**0.93±0.68**
	16					5								1	**1.95±0.80**	2.20±0.66	1.17±0.87	1.75±1.54	**0.58±0.25**	0.69±0.42

Neural Network estimates based on different sensor locations and numbers. The sensor number and color correspond to the right human figure (extracted from Fig. 1). The RMSEs for the ML, AP, and IS respectively are from the two testing groups. Bolded numbers indicated a significant difference with at least one other sensor set. Significance level is indicated in parenthesis for each group (*p=0.05).

**TABLE IV:** RMSEs for Pathological Gait Estimates

CoM Profile	Set 1 (cm)	Set 6 (cm)	Set 9 (cm)	Set 13 (cm)

ML	3.23 ± 0.81	3.45 ± 0.62	4.19 ± 0.59	2.91 ± 0.62
AP	1.77 ± 0.45	1.96 ± 0.48	3.33 ± 1.35	1.77 ± 0.62
IS	0.79 ± 0.20	1.16 ± 0.26	1.26 ± 0.45	1.35 ± 0.39
